# Risk of Dementia in Patients With Type 2 Diabetes Using SGLT2 Inhibitors Versus DPP‐4 Inhibitors: A Systematic Review and Meta‐Analysis

**DOI:** 10.1002/edm2.70174

**Published:** 2026-03-03

**Authors:** Kiran Kumari, Anusha Bai, Fnu Geeta, Nirmal Wadhwani, Rohit Kumar, Ajay Kumar, Radhika Kumari, Lata Bai, Fnu Muskan, Fnu Kashish, Mohammed Yousafzai

**Affiliations:** ^1^ Ziauddin Medical University Karachi Pakistan; ^2^ Chandka Medical College Larkana Pakistan; ^3^ Bahria University Health Sciences Campus Karachi Pakistan; ^4^ Bahria University Medical and Dental College Karachi Pakistan; ^5^ Shaheed Mohtarma Benazir Bhutto Medical College, Lyari Karachi Pakistan; ^6^ Peoples University of Medical and Health Sciences for Women Nawabshah Pakistan; ^7^ Gambat Institute of Medical Sciences Gambat Pakistan; ^8^ Kabul University of Medical Sciences Kabul Afghanistan

## Abstract

**Background:**

Type 2 diabetes mellitus (T2DM) is a known risk factor for dementia, yet the cognitive impact of different glucose‐lowering therapies remains unclear. Emerging evidence suggests sodium‐glucose cotransporter‐2 (SGLT2) inhibitors may confer neuroprotective benefits compared to dipeptidyl peptidase‐4 (DPP‐4) inhibitors.

**Objective:**

To compare the risk of incident dementia among patients with T2DM initiating SGLT2 inhibitors versus DPP‐4 inhibitors.

**Methods:**

A systematic search of PubMed, Scopus, and the Cochrane Central Register of Controlled Trials was conducted through June 1, 2025. The primary outcome was all‐cause dementia. Secondary outcomes included Alzheimer's disease and vascular dementia. Random‐effects models were used to pool adjusted hazard ratios (HRs), and subgroup analyses explored heterogeneity by age, sex, and specific SGLT2 agents.

**Results:**

Nine retrospective cohort studies encompassing 2,433,086 individuals (601,692 SGLT2i users; 1,831,394 DPP‐4i users) met inclusion criteria. SGLT2 inhibitors were associated with a significantly lower risk of all‐cause dementia (HR = 0.74; 95% CI: 0.62–0.87), Alzheimer's disease (HR = 0.62; 95% CI: 0.52–0.74), and vascular dementia (HR = 0.54; 95% CI: 0.49–0.60) compared to DPP‐4 inhibitors. Subgroup findings were largely consistent across age and sex. Dapagliflozin and empagliflozin showed significant benefit, while canagliflozin did not.

**Conclusion:**

Use of SGLT2 inhibitors is significantly associated with lower dementia risk compared to DPP‐4 inhibitors in patients with T2DM. Prospective trials are warranted to confirm these findings and explore underlying mechanisms.

## Graphical abstract

SGLT2 inhibitors were associated with a significantly lower risk of incident dementia compared with DPP‐4 inhibitors in patients with type 2 diabetes. This meta‐analysis synthesises evidence from diverse real‐world cohorts and highlights the potential neuroprotective benefits of SGLT2 inhibition beyond glycemic control.

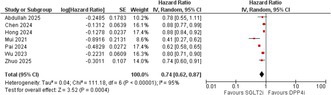

## 1 Introduction

Dementia, a progressive neurodegenerative disorder characterised by cognitive decline and loss of functional independence, poses a substantial and growing public health burden. With an estimated 55 million individuals affected globally and projected costs exceeding $1 trillion annually in 2019, the urgency for effective prevention strategies continues to intensify [1, 2]. Among the established modifiable risk factors, type 2 diabetes mellitus (T2DM) has consistently been associated with a significantly elevated risk of both Alzheimer's disease and vascular dementia [3, 4]. This association is underpinned by shared pathophysiological mechanisms such as insulin resistance, oxidative stress, chronic inflammation, and microvascular dysfunction, pathways similarly implicated in the aetiology of Parkinson's disease [5, 6].

The advent of novel glucose‐lowering therapies has reshaped diabetes management, offering benefits beyond glycemic control [7]. Sodium‐glucose cotransporter‐2 (SGLT2) inhibitors, in particular, have demonstrated compelling cardiovascular and renal protective effects in large randomised trials [8]. Emerging preclinical and clinical data suggest they may also confer neuroprotective benefits through mechanisms such as improved cerebral energy metabolism, blood–brain barrier stabilisation, and attenuation of neuroinflammation [9]. Conversely, dipeptidyl peptidase‐4 (DPP‐4) inhibitors, another widely prescribed second‐line class, have shown largely neutral effects on cognitive outcomes in randomised settings [10, 11].

While a growing body of observational studies has compared the cognitive effects of these two drug classes, findings have been mixed and methodology varies across studies [12, 13, 14, 15]. Several have reported a lower incidence of dementia with SGLT2 inhibitors compared to DPP‐4 inhibitors, whereas others have shown age‐ and molecule‐specific discrepancies. For example, Shin et al. reported a 35% lower dementia risk among younger adults using SGLT2i, while Abdullah et al. found no significant difference overall between SGLT2i and DPP‐4i users, with the association reaching significance only in older adults [12, 15]. Similarly, agent‐specific results have varied. Dapagliflozin and empagliflozin showing protective effects in some cohorts, whereas canagliflozin has demonstrated neutral associations [16, 17]. These inconsistencies underscore the need for a harmonised quantitative synthesis.

A 2020 Bayesian network meta‐analysis compared multiple antidiabetic classes for dementia risk and identified DPP‐4 inhibitors as the agents most strongly associated with lower dementia incidence relative to other therapies and non‐use [18]. However, SGLT2 inhibitors were not evaluated in that analysis due to insufficient data at the time. This omission underscores the need for updated comparative evidence as SGLT2 inhibitor data have since expanded substantially.

To address this gap, we conducted a comprehensive meta‐analysis to quantify the relative risk of incident dementia in patients with T2DM initiating SGLT2 inhibitors versus DPP‐4 inhibitors. We also aimed to explore whether this association differs by dementia subtype (e.g., Alzheimer's disease, vascular dementia), patient demographics (e.g., age, sex), or specific SGLT2 agents. By synthesising available evidence, our study aims to support clinical decision‐making and guide future research in dementia prevention within diabetes care.

## 2 Methods

This study was conducted in accordance with the Cochrane Handbook for Systematic Reviews of Interventions and reported following the Preferred Reporting Items for Systematic Reviews and Meta‐Analyses (PRISMA) guidelines [19]. The study protocol was registered in PROSPERO (ID: 420251071432).

### 2.1 Eligibility Criteria and Outcomes

Eligible studies included randomised controlled trials as well as prospective or retrospective cohort studies involving adults (≥ 18 years) with T2DM who initiated treatment with an SGLT2 inhibitor and were directly compared with a DPP‐4 inhibitor group. Studies were required to report adjusted hazard ratios (HRs) comparing the incidence of dementia between the two drug classes, along with corresponding 95% confidence intervals (CIs). The primary outcome was incident all‐cause dementia, defined as the first recorded clinical diagnosis during follow‐up. Secondary outcomes included incidence of Alzheimer's disease and vascular dementia. Subgroup analyses were conducted for the primary outcome to examine effect modification by age (< 65 vs. ≥ 65 years), sex (male vs. female), and individual SGLT2 inhibitors (e.g., dapagliflozin, empagliflozin, canagliflozin).

Studies were excluded if they (1) did not involve human participants; (2) were single‐arm or cross‐sectional in design; or (3) were reviews, case reports, or case series.

### 2.2 Data Sources and Search Strategy

We performed a comprehensive literature search across PubMed, Scopus, and the Cochrane Central Register of Controlled Trials from inception to 1 June 2025. Search terms combined controlled vocabulary and free‐text keywords related to SGLT2 inhibitors, DPP‐4 inhibitors, and dementia or cognitive impairment using Boolean operators. The full search strategy is provided in Table S1. After de‐duplication using Rayyan.ai, two independent reviewers (KK and AB) screened titles and abstracts, followed by full‐text reviews based on predefined eligibility criteria. Disagreements were resolved through consensus.

### 2.3 Data Extraction and Quality Assessment

Two reviewers (KK and AB) independently extracted data and assessed methodological quality. Extracted information included study design, sample characteristics, and outcome measures. Data were recorded in a standardised Excel sheet. When multiple studies drew outcomes from the same database with overlapping periods, the study with the larger sample size was included. Risk of bias was evaluated using tools appropriate to study design. The Cochrane Risk of Bias 2 (RoB 2) tool was planned for randomised controlled trials (RCTs), and the Newcastle–Ottawa Scale (NOS) for observational studies. However, no eligible RCTs were identified in the final dataset; therefore, only the NOS was applied.

The NOS evaluates three major domains: selection of study cohorts (representativeness of the exposed cohort, selection of the non‐exposed group, ascertainment of exposure, and confirmation that the outcome was not present at baseline), comparability of cohorts (control for key confounding factors such as age, sex, and major comorbidities), and outcome assessment (objectivity and adequacy of outcome ascertainment, follow‐up duration, and completeness of follow‐up).

Each item is assigned one point, with a maximum total score of nine. Studies scoring 7–9 points were considered high quality, 5–6 points as moderate quality, and ≤ 4 points as low quality. Discrepancies in scoring between reviewers were resolved by discussion and consensus.

All authors reviewed extracted data for accuracy and completeness.

### 2.4 Statistical Analysis

The primary summary measure was the adjusted hazard ratio (HR) with 95% confidence intervals [20]. Because all included studies reported time‐to‐event outcomes with multivariable adjustment, effect estimates were pooled using the Generic Inverse Variance method, which enables synthesis of log‐transformed adjusted HRs and standard errors while preserving each study's covariate adjustment. A random‐effects model (DerSimonian–Laird) was selected a priori, as meaningful clinical and methodological heterogeneity was anticipated across studies, including variations in population demographics, healthcare setting, dementia ascertainment, exposure definitions, and covariate adjustment [21]. A fixed‐effect model was therefore deemed inappropriate for this evidence base. Heterogeneity was assessed using the Higgins *I* ^2^ statistic, with *I* ^2^ ≤ 50% indicating acceptable variability [22, 23, 24]. A *p*‐value < 0.05 was considered statistically significant. Forest plots were used to visualise effect estimates. Formal assessment of publication bias using funnel plots was not conducted due to insufficient statistical power when fewer than 10 studies are available [25]. Sensitivity analyses were performed by excluding one study at a time to test the robustness of results. All analyses were conducted using Review Manager (RevMan) version 5.4 (Copenhagen: The Nordic Cochrane Centre, The Cochrane Collaboration, 2020).

## 3 Results

### 3.1 Literature Search and Selection

**FIGURE 1:**
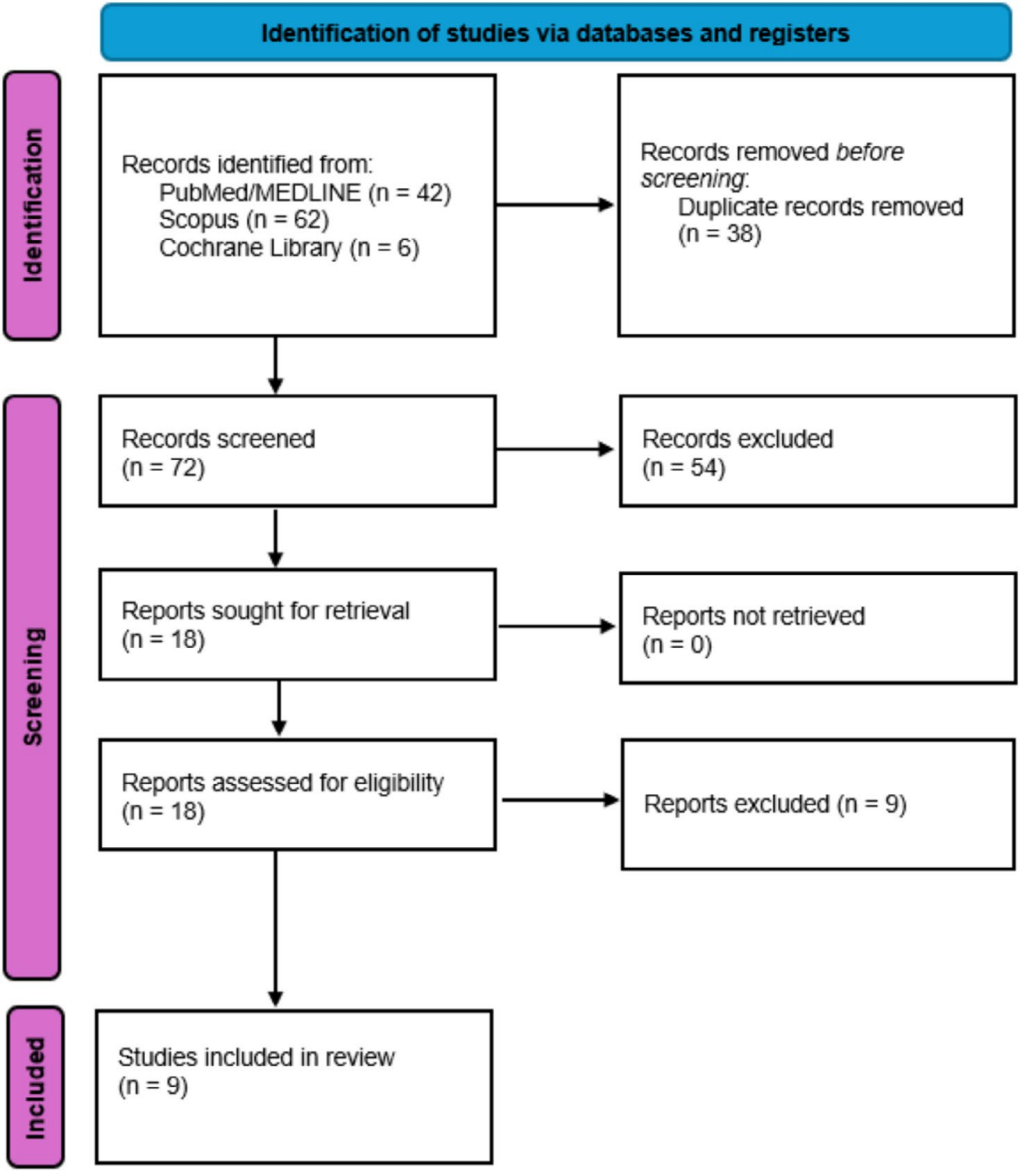
PRISMA flowchart.

The initial database search identified 110 records. After removing duplicates, 72 unique titles and abstracts were screened. Of these, 18 articles were selected for full‐text review based on predefined eligibility criteria. Nine studies met the inclusion criteria and were included in the final meta‐analysis [12, 13, 14, 15, 16, 17, 26, 27, 28]. A detailed overview of the screening and selection process is presented in Figure 1.

### 3.2 Study Characteristics

All nine studies included in the meta‐analysis were retrospective observational cohorts published between 2021 and 2025. Collectively, these studies encompassed a total of 2,433,086 patients, including 601,692 treated with SGLT2 inhibitors and 1,831,394 with DPP‐4 inhibitors. The cohorts were drawn from diverse geographic regions, including Canada, Hong Kong, South Korea, China, Taiwan, and the United Kingdom. Across studies, patients initiating SGLT2 inhibitors tended to be slightly younger (mean age range: 56.8–72.4 years) than those prescribed DPP‐4 inhibitors (61.8–74.3 years). Additionally, the proportion of female participants was consistently lower in the SGLT2i groups. Detailed baseline characteristics of the included studies are summarised in Table 1 and Table S4. A detailed summary of the adjustment factors included in the multivariable models of each study is provided in Table S3.

**TABLE 1:** Baseline characteristics of included studies.

Study	Country	Study design	Data source	N (SGLT2i)	N (DPP‐4i)	Mean Age (SGLT2i)	Mean Age (DPP‐4i)	Female (%) SGLT2i	Female (%) DPP‐4i	Follow‐up (years)
Wu et al. (2023) [16]	Canada	Retrospective cohort	Ontario administrative databases	36,513	70,390	72.4	74.3	38.8	47	2.8
Mui et al. (2021) [26]	Hong Kong	Retrospective cohort	Clinical Data Analysis and Reporting System	13,276	36,544	61.17	68.38	38.01	46.64	1.3
Hong et al. (2024) [27]	South Korea	Retrospective cohort	National Health Insurance Service‐National Health Insurance Database	42,874	385,045	59.8	64.7	58.7	56.2	4.8
Zhuo et al. (2025) [14]	China	Retrospective cohort	Yinzhou Regional Health Care Database	19,931	27,404	65.2	65.4	43.9	47.5	1.6 (SGLT2i), 2.8 (DPP‐4i)
Chen et al. (2024) [28]	Taiwan	Retrospective cohort	National Health Insurance Research Database	810	902	65.9	—	43	—	Up to 5
Pai et al. (2024) [13]	Global	Retrospective cohort	TriNetX	237,100	244,834	63.25	65.49	40.33	49.59	1.9 (SGLT2i), 3.3 (DPP‐4i)
Abdullah et al. (2025) [12]	UK	Retrospective cohort	Clinical Practice Research Datalink Aurum	34,816	83,190	56.83	62.34	39	40	1.5 (SGLT2i), 1.8 (DPP‐4i)
Liu et al. (2025) [17]	Global	Retrospective cohort	TriNetX	103,709	135,086	69.9	70	39.2	51	Up to 5
Shin et al. (2024) [15]	South Korea	Retrospective cohort	National Health Insurance Service database	112,663	847,999	61.9	61.8	44.2	41.1	1.8

Abbreviations: DPP‐4i, dipeptidyl peptidase‐4 inhibitor; N, number of participants; SGLT2i, sodium‐glucose cotransporter‐2 inhibitor; UK, United Kingdom.

Quality assessment using the Newcastle‐Ottawa Scale indicated high methodological rigour for all included studies, each scoring the maximum of 9 out of 9 stars (Table S2). Publication bias was not assessed because the number of included studies (*n* = 9) was below the recommended threshold for reliable funnel‐plot interpretation [25].

### 3.3 Results of Meta‐Analysis

#### 3.3.1 Incident All‐Cause Dementia

**FIGURE 2:**
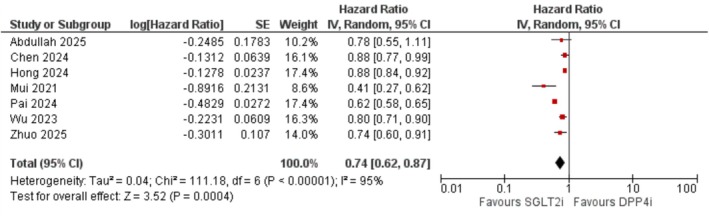
Forest plot of the association between SGLT2 inhibitor use and risk of all‐cause dementia compared to DPP‐4 inhibitors.

SGLT2 inhibitor use was associated with a significantly lower risk of incident dementia compared to DPP‐4 inhibitors (HR = 0.74; 95% CI: 0.62–0.87; *I* ^2^ = 95%; *p* < 0.001) (Figure 2). Sensitivity analyses revealed no significant impact on effect size or heterogeneity upon exclusion of individual studies.

#### 3.3.2 Alzheimer's Disease

**FIGURE 3:**
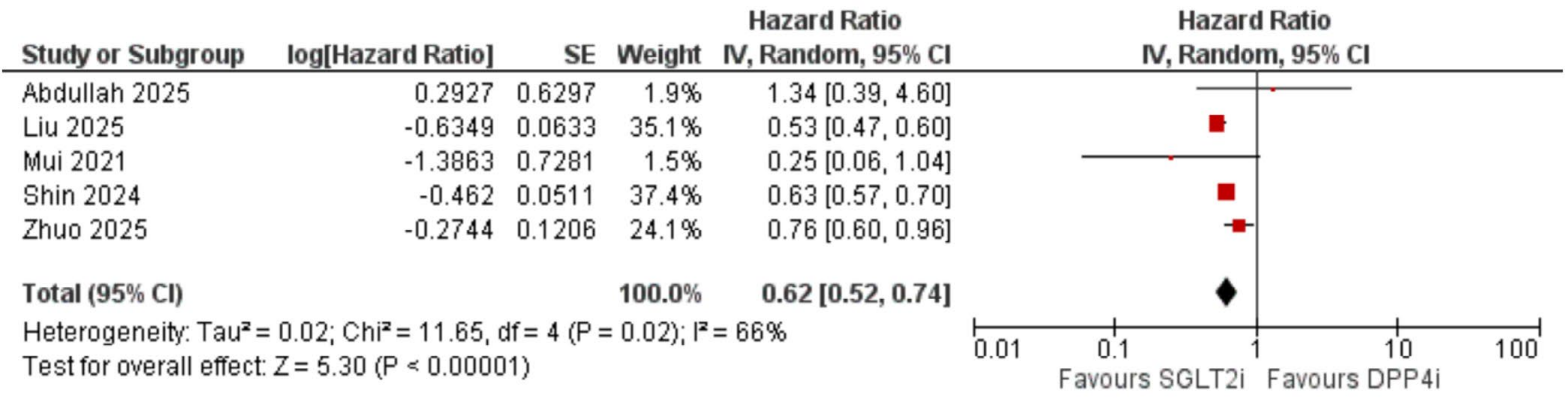
Forest plot showing the effect of SGLT2 inhibitors versus DPP‐4 inhibitors on the risk of Alzheimer's disease.

The risk of Alzheimer's disease was significantly reduced in the SGLT2i group (HR = 0.62; 95% CI: 0.52–0.74; *I* ^2^ = 66%; *p* < 0.001) (Figure 3). Excluding Liu et al. from the analysis reduced heterogeneity (*I* ^2^ = 41%), with a consistent effect estimate (HR = 0.68; 95% CI: 0.55–0.83; *p* < 0.001) (Figure S1).

#### 3.3.3 Vascular Dementia

**FIGURE 4:**
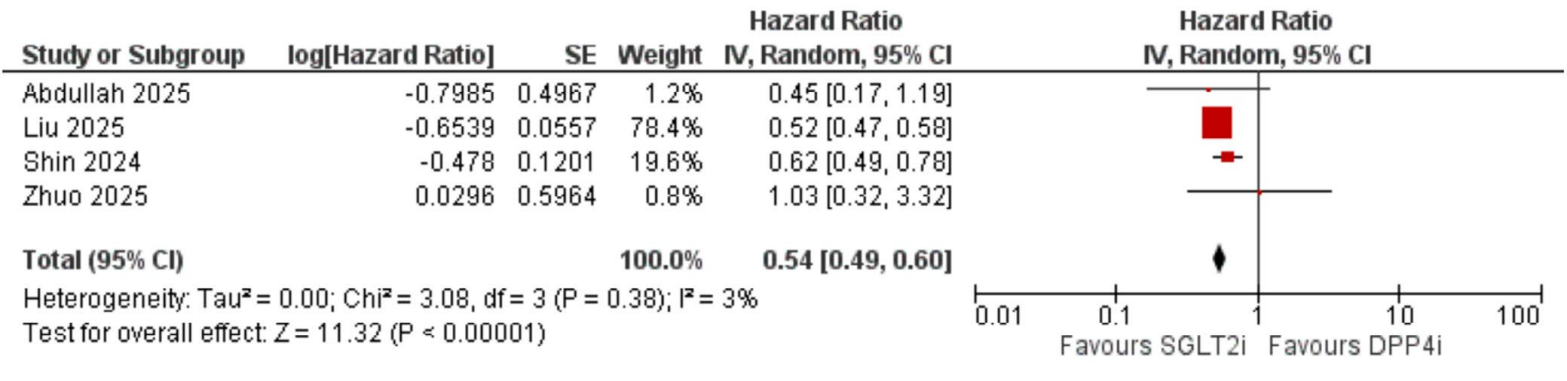
Forest plot illustrating the comparative risk of vascular dementia in patients treated with SGLT2 inhibitors versus DPP‐4 inhibitors.

SGLT2 inhibitors demonstrated a strong protective association against vascular dementia (HR = 0.54; 95% CI: 0.49–0.60; *I* ^2^ = 3%; *p* < 0.001), with minimal heterogeneity (Figure 4).

#### 3.3.4 Subgroup Analyses for Incident All‐Cause Dementia

##### 3.3.4.1 By Age

**FIGURE 5:**
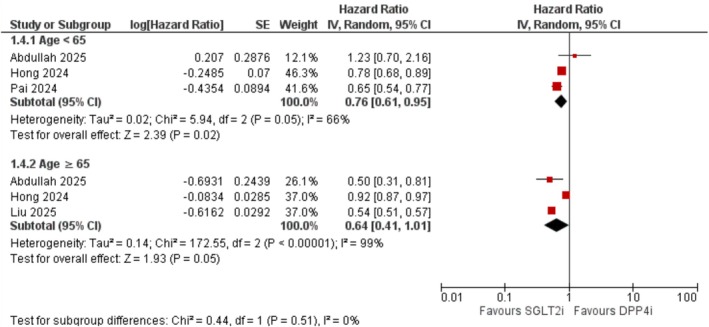
Subgroup forest plot of all‐cause dementia stratified by age group.

Among individuals aged < 65 years, SGLT2 inhibitors were associated with a significantly lower dementia risk (HR = 0.76; 95% CI: 0.61–0.95; *I* ^2^ = 66%; *p* = 0.02). In patients aged ≥ 65 years, a non‐significant trend was observed (HR = 0.64; 95% CI: 0.41–1.01; *I* ^2^ = 99%; *p* = 0.05) (Figure 5).

##### 3.3.4.2 By Sex

**FIGURE 6:**
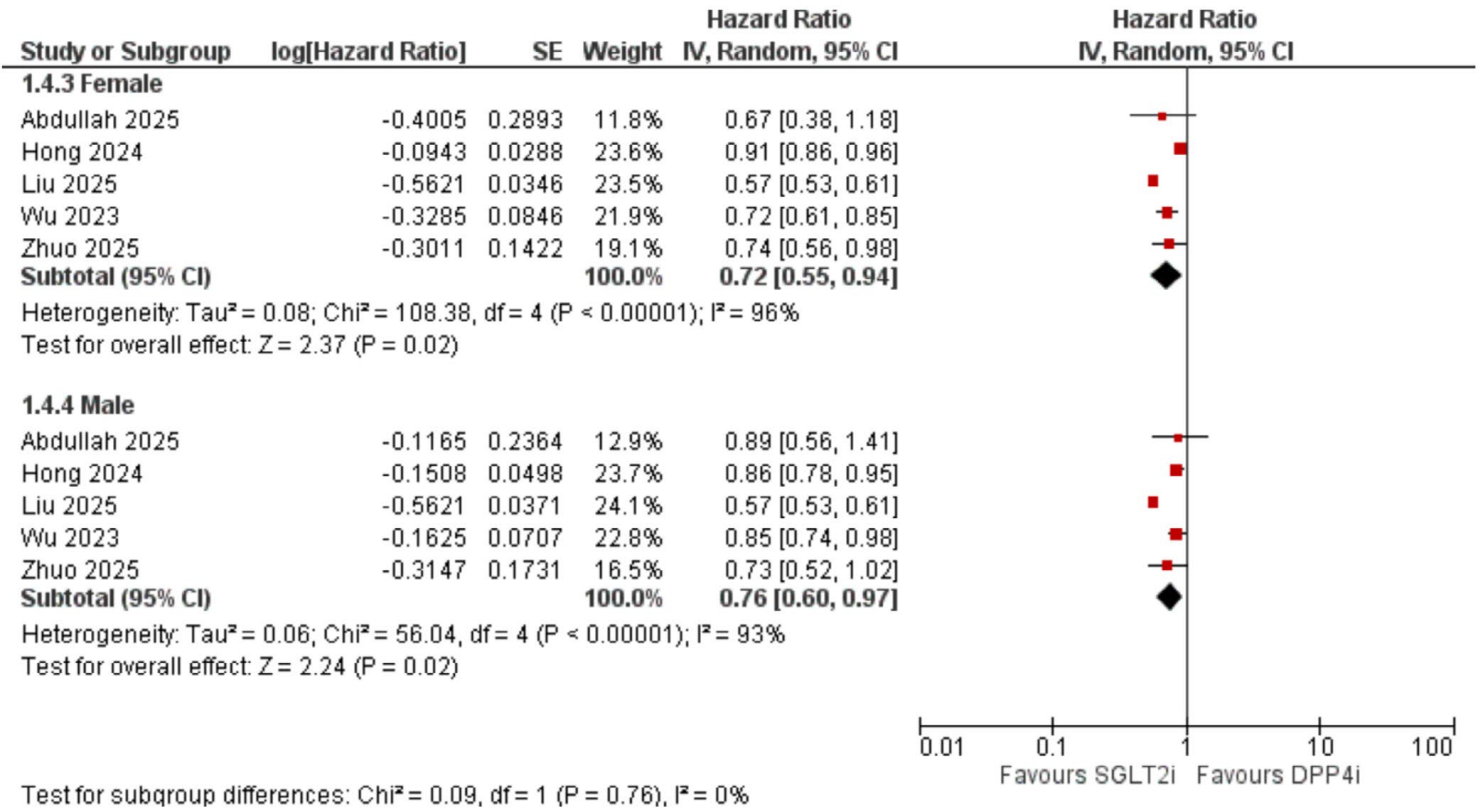
Subgroup forest plot of all‐cause dementia stratified by sex.

SGLT2 inhibitors were protective in both sexes. In females, the pooled HR was 0.72 (95% CI: 0.55–0.94; *I* ^2^ = 96%; *p* = 0.02), and in males, it was 0.76 (95% CI: 0.60–0.97; *I* ^2^ = 93%; *p* = 0.02) (Figure 6).

##### 3.3.4.3 By SGLT2 Inhibitor Type

**FIGURE 7:**
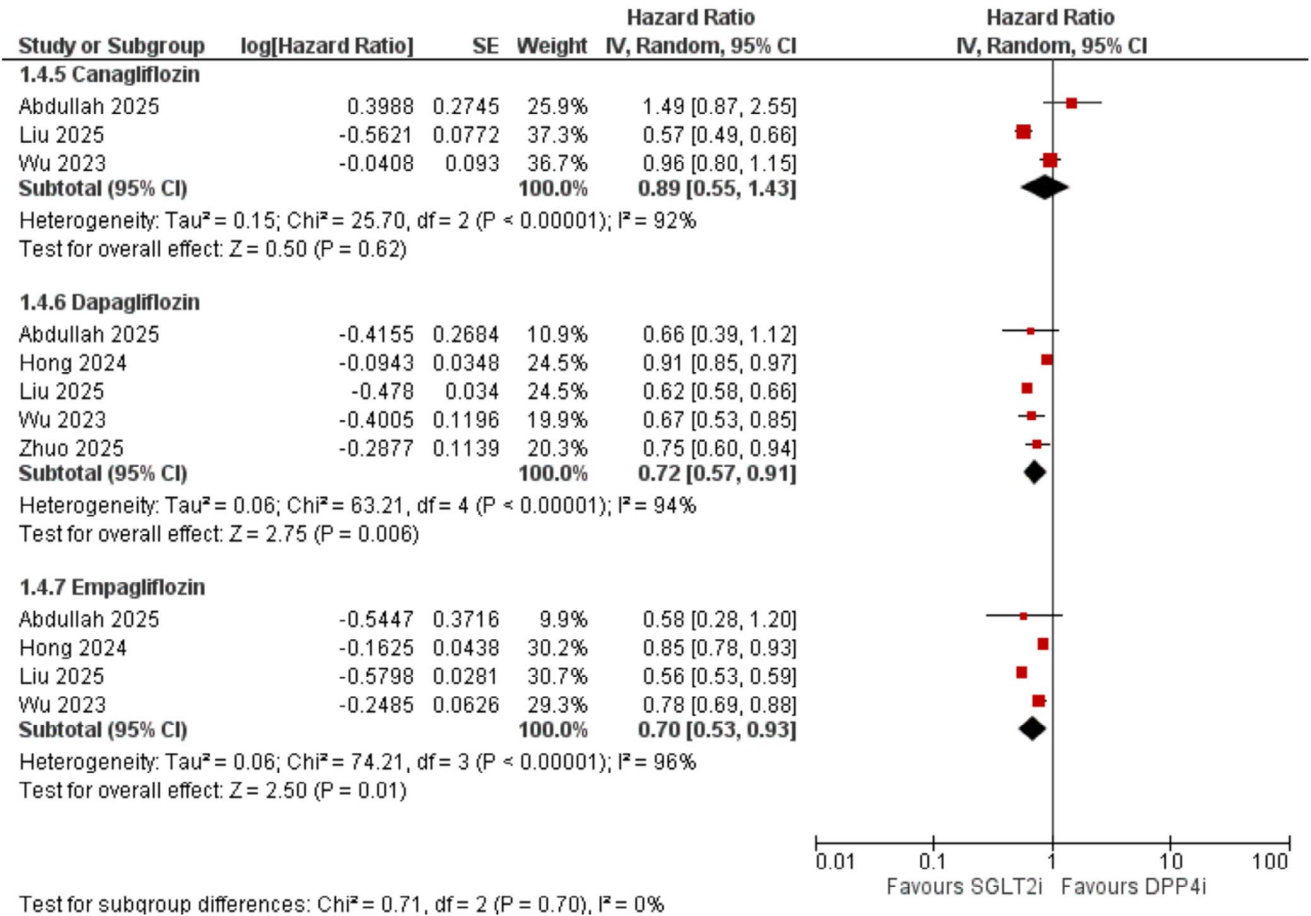
Subgroup forest plot of all‐cause dementia stratified by individual SGLT2 inhibitor agent.

Dapagliflozin (HR = 0.72; 95% CI: 0.57–0.91; *I* ^2^ = 94%; *p* = 0.006) and empagliflozin (HR = 0.70; 95% CI: 0.53–0.93; *I* ^2^ = 96%; *p* = 0.01) were both significantly associated with reduced dementia risk. Canagliflozin showed no significant association (HR = 0.89; 95% CI: 0.55–1.43; *I* ^2^ = 92%; *p* = 0.62) (Figure 7).

## 4 Discussion

This meta‐analysis indicates that SGLT2 inhibitors are significantly associated with a reduced risk of incident dementia in patients with T2DM, including both Alzheimer's and vascular dementia. These findings were consistent across age, sex, and specific agents such as dapagliflozin and empagliflozin.

Our findings add to a growing but heterogeneous body of literature examining the relationship between glucose‐lowering therapies and cognitive outcomes. Prior studies have explored the impact of various antidiabetic agents, including metformin, insulin, sulfonylureas, and GLP‐1 receptor agonists, on dementia risk, with mixed results [29, 30]. A previous network meta‐analysis ranked DPP‐4 inhibitors among the most protective agents against dementia but did not include SGLT2 inhibitors owing to limited data at the time [18]. Subsequent work has suggested that SGLT2 inhibitor use is associated with lower dementia risk compared with nonuse, but these analyses relied on placebo or standard‐care comparators and therefore did not clarify how SGLT2 inhibitors perform relative to other active antidiabetic therapies [31]. A recent meta‐analysis evaluating SGLT2 inhibitors against nonusers reported a significantly reduced risk of dementia, which is similar in direction and magnitude to our findings; however, that study did not assess comparative effects across antidiabetic drug classes [32].

Similarly, a recent meta‐analysis of DPP‐4 inhibitors reported improvements in cognitive impairment scores but focused on short‐term neurocognitive testing rather than long‐term dementia incidence, limiting its relevance to disease‐modifying effects [33]. An additional broad synthesis of newer glucose‐lowering drugs, including SGLT2 inhibitors, DPP‐4 inhibitors, and GLP‐1 receptor agonists, reported reduced dementia risk across multiple classes but did not provide direct comparisons between them and offered limited data for Alzheimer's and vascular dementia subtypes [31].

Taken together, prior research has highlighted potential neuroprotective effects across several drug classes yet has lacked a direct evaluation of the comparative cognitive impact of SGLT2 versus DPP‐4 inhibitors. By consolidating data from large, diverse observational cohorts across multiple health systems, the present meta‐analysis addresses this critical evidence gap. To our knowledge, it provides the first comprehensive head‐to‐head synthesis of these two widely used second‐line therapies, offering clinically relevant insight into their relative associations with dementia risk.

The biological plausibility of neuroprotection with SGLT2 inhibitors is well‐supported. These agents exhibit anti‐inflammatory and antioxidant properties, which mitigate key drivers of dementia pathology [9]. Preclinical studies have shown that empagliflozin reduces oxidative stress and preserves blood–brain barrier integrity in diabetic models [34, 35]. These vascular benefits are likely to enhance cerebral perfusion and reduce the risk of vascular cognitive impairment. These benefits align with broader evidence from diabetes populations showing that optimised cardiovascular risk reduction meaningfully lowers vascular complications [36].

SGLT2 inhibitors also induce mild ketosis, elevating levels of neuroprotective ketones such as β‐hydroxybutyrate, which support mitochondrial function and synaptic plasticity [9, 37]. Additionally, they appear to modulate amyloid processing and reduce acetylcholinesterase activity in preclinical Alzheimer's models, suggesting potential disease‐modifying effects [9].

By contrast, DPP‐4 inhibitors primarily enhance GLP‐1 signalling but do not exhibit the broader metabolic, vascular, or neuroprotective effects observed with SGLT2 inhibitors. Although some preclinical studies suggest that DPP‐4 inhibition may attenuate blood–brain barrier disruption, oxidative stress, and neuroinflammation, these effects have not consistently translated into meaningful cognitive improvements in clinical trials [10, 11, 38, 39]. The CARMELINA‐COG substudy, conducted in patients with type 2 diabetes and high cardiorenal risk, found no significant difference in the incidence of accelerated cognitive decline between linagliptin and placebo over a median follow‐up of 2.5 years (OR 0.96; 95% CI: 0.77–1.19) [10]. Similarly, the CAROLINA‐COGNITION trial, which compared linagliptin to glimepiride in individuals with relatively early‐stage type 2 diabetes and elevated cardiovascular risk, reported no difference in cognitive outcomes over 6.1 years (OR 1.01; 95% CI: 0.86–1.18) [11]. No significant differences were observed in subgroup analyses. These findings support the conclusion that, despite some mechanistic plausibility from animal models, DPP‐4 inhibitors lack consistent neurocognitive benefit in clinical practice.

Subgroup analyses in our meta‐analysis revealed that the protective association of SGLT2 inhibitors was generally consistent across age and sex strata. While the effect in older adults (≥ 65 years) did not reach statistical significance, the confidence interval narrowly crossed unity, suggesting the possibility of insufficient statistical power rather than a true null effect. Notably, dapagliflozin and empagliflozin were each associated with significantly reduced dementia risk, whereas canagliflozin showed no significant association. The reason for this discrepancy remains uncertain but may reflect pharmacologic differences among the agents. Of the three most widely used SGLT2 inhibitors, canagliflozin exhibits the lowest selectivity for SGLT2 over SGLT1 [40]. Although the underlying mechanisms remain speculative, it is plausible that more selective inhibition of SGLT2 may confer greater neuroprotective effects. This hypothesis warrants further investigation in mechanistic and comparative studies.

To confirm these associations, prospective RCTs with dementia or cognitive decline as primary endpoints are urgently needed. Such studies should include neuroimaging, fluid biomarkers, and cognitive testing. Research should also evaluate potential mechanisms, population‐specific effects, cost‐effectiveness, and the optimal timing and duration of SGLT2 inhibitor use for dementia prevention.

These findings may have important implications for clinical practice. Given the consistent association between SGLT2 inhibitor use and lower dementia risk across multiple subgroups and dementia subtypes, clinicians may consider prioritising SGLT2 inhibitors in patients with type 2 diabetes who are at elevated risk for cognitive decline, particularly when cardiovascular or renal protection is also desired. While DPP‐4 inhibitors remain a well‐tolerated, neutral option, especially in frail or older adults, our results suggest they may not offer comparable neurocognitive benefits. Nonetheless, treatment decisions should remain individualised, accounting for comorbidities, tolerability, and contraindications. Importantly, the observational nature of available evidence warrants caution, and definitive guidance will depend on future randomised trials incorporating cognitive endpoints.

However, limitations warrant consideration. The analysis relies entirely on observational data, which, despite propensity adjustment and robust matching, cannot fully eliminate residual confounding or channelling bias. Dementia diagnoses were typically based on administrative coding, potentially leading to underreporting or misclassification. Additionally, the relatively short follow‐up period in certain studies may have constrained the ability to detect long‐term outcomes associated with dementia, given the chronic and progressive nature of the condition. Finally, these findings remain observational in nature and causal inference cannot be definitively established.

## 5 Conclusion

This meta‐analysis demonstrates a significant association between SGLT2 inhibitor use and reduced risk of dementia, including Alzheimer's and vascular subtypes, when compared to DPP‐4 inhibitors in patients with T2DM. Benefits were consistent across demographic subgroups and specific SGLT2 agents, such as dapagliflozin and empagliflozin. These findings support the potential role of SGLT2 inhibitors in preserving cognitive function in diabetes management. Further high‐quality randomised trials are needed to validate these associations and clarify underlying mechanisms.

## Author Contributions

**Kiran Kumari:** conceptualisation (lead), methodology (lead), data curation (lead), formal analysis (lead), visualisation (lead), writing – original draft (lead), project administration (lead), writing – review and editing (equal); **Anusha Bai:** methodology (equal), data curation (equal), study screening (equal), quality assessment (equal), writing – original draft (supporting), writing – review and editing (equal); **Fnu Geeta:** data curation (supporting), quality assessment (supporting), writing – review and editing (equal); **Nirmal Wadhwani:** writing – review and editing (equal), interpretation of results (supporting); **Rohit Kumar:** validation (supporting), writing – review and editing (equal), interpretation of findings (supporting); **Ajay Kumar:** writing – review and editing (equal), clinical interpretation (supporting); **Radhika Kumari:** writing – review and editing (equal), conceptualisation (supporting); **Lata Bai:** data presentation (supporting), writing – review and editing (equal); **Fnu Muskan:** organisation of supporting material (supporting), writing – review and editing (equal); **Fnu Kashish:** figure preparation (supporting), writing – review and editing (equal); **Mohammed Yousafzai:** conceptualisation (lead), methodological oversight (lead), supervision (lead), writing – review and editing (equal), final approval (lead).

## Funding

The authors have nothing to report.

## Ethics Statement

The authors have nothing to report.

## Conflicts of Interest

The authors declare no conflicts of interest.

## Supporting information


**Table S1:** Detailed search strategy used in each database.
**Table S2:** Quality assessment of included cohort studies using the Newcastle‐Ottawa Scale (NOS).
**Table S3:** Key adjustment factors and covariates.
**Table S4:** Baseline prevalence of key dementia risk factors.
**Figure S1:** *S*ensitivity analysis for the association between SGLT2 inhibitor use and risk of Alzheimer's disease after exclusion of Liu et al.

## Data Availability

Data sharing not applicable to this article as no datasets were generated or analysed during the current study.
