# Supplementary material for: Risk of Dementia in Patients With Type 2 Diabetes Using SGLT2 Inhibitors Versus DPP‐4 Inhibitors: A Systematic Review and Meta‐Analysis
Source: Endocrinol Diabetes Metab. 2026 Mar 3;9(2):e70174. doi: 10.1002/edm2.70174 (PMC12957778; doi:10.1002/edm2.70174)

**Supplementary file**

**Supplementary Table 1:** Detailed search strategy used in each database

| **Database** | **String** | **Results** |
| --- | --- | --- |
| PubMed | ("SGLT2 inhibitors" OR "sodium-glucose cotransporter 2 inhibitors" OR SGLT2i OR canagliflozin OR dapagliflozin OR empagliflozin OR ertugliflozin)  AND  ("DPP-4 inhibitors" OR "dipeptidyl peptidase 4 inhibitors" OR "dipeptidyl peptidase IV inhibitors" OR DPP-4i OR DPP-IV OR sitagliptin OR linagliptin OR saxagliptin OR alogliptin OR vildagliptin)  AND  (dementia OR "cognitive decline" OR "cognitive impairment" OR "Alzheimer's disease" OR "vascular dementia" OR "neurocognitive disorders" OR "time to dementia") | 42 |
| Cochrane | ("SGLT2 inhibitors" OR "sodium-glucose cotransporter 2 inhibitors" OR SGLT2i OR canagliflozin OR dapagliflozin OR empagliflozin OR ertugliflozin)  AND  ("DPP-4 inhibitors" OR "dipeptidyl peptidase 4 inhibitors" OR "dipeptidyl peptidase IV inhibitors" OR DPP-4i OR DPP-IV OR sitagliptin OR linagliptin OR saxagliptin OR alogliptin OR vildagliptin)  AND  (dementia OR "cognitive decline" OR "cognitive impairment" OR "Alzheimer's disease" OR "vascular dementia" OR "neurocognitive disorders" OR "time to dementia") | 6 |
| Scopus | TITLE-ABS-KEY("SGLT2 inhibitors" OR "sodium-glucose cotransporter 2 inhibitors" OR SGLT2i OR canagliflozin OR dapagliflozin OR empagliflozin OR ertugliflozin)  AND  TITLE-ABS-KEY("DPP-4 inhibitors" OR "dipeptidyl peptidase 4 inhibitors" OR "dipeptidyl peptidase IV inhibitors" OR DPP-4i OR DPP-IV OR sitagliptin OR linagliptin OR saxagliptin OR alogliptin OR vildagliptin)  AND  TITLE-ABS-KEY(dementia OR "cognitive decline" OR "cognitive impairment" OR "Alzheimer's disease" OR "vascular dementia" OR "neurocognitive disorders" OR "time to dementia") | 62 |

**Supplementary Table 2:** Quality Assessment of Included Cohort Studies Using the Newcastle-Ottawa Scale (NOS).

| **Domain** | **Selection** | | | | **Comparability** | **Outcomes** | | | **Verdict** | |
| --- | --- | --- | --- | --- | --- | --- | --- | --- | --- | --- |
| **Study Name** | **Representativeness of the exposed cohort** | **Selection of the non-exposed cohort** | **Ascertainment of exposure** | **Demonstration that outcome of interest was not present at start of study** | **Comparability of cohorts based on the design or analysis** | **Assessment of outcome** | **Was follow-up long enough for outcomes to occur** | **Adequacy of follow-up of cohorts** | **Total Score** | **Good/Fair/Poor** |
| Wu et al. (2023) | * | * | * | * | ** | * | * | * | 9 | Good |
| Mui et al. (2021) | * | * | * | * | ** | * | * | * | 9 | Good |
| Hong et al. (2024) | * | * | * | * | ** | * | * | * | 9 | Good |
| Zhuo et al. (2025) | * | * | * | * | ** | * | * | * | 9 | Good |
| Chen et al. (2024) | * | * | * | * | ** | * | * | * | 9 | Good |
| Pai et al. (2024) | * | * | * | * | ** | * | * | * | 9 | Good |
| Abdullah et al. (2025) | * | * | * | * | ** | * | * | * | 9 | Good |
| Liu et al. (2025) | * | * | * | * | ** | * | * | * | 9 | Good |
| Shin et al. (2024) | * | * | * | * | ** | * | * | * | 9 | Good |

**Supplementary Table 3:** Key Adjustment Factors and Covariates

.

| **Study** | **Adjustment Method(s)** | **Key Adjustment Factors (Covariates)** |
| --- | --- | --- |
| Wu et al. (2023) | Inverse Probability Treatment Weighting (IPTW) | Age, sex, rural residence, diabetes duration, polypharmacy (number of chronic drugs), comorbidities (e.g., CKD, AF/flutter, CHF, obesity, hypertension, stroke), glucose-lowering medications (e.g., Insulin, Metformin, Sulfonylureas, GLP-1RA, Thiazolidinediones), and other concomitant medications/proxy variables (e.g., ACEi/ARB, beta blockers, statins, diuretics, antiplatelets, antidepressants, healthcare utilization, investigation frequencies) |
| Mui et al. (2021) | Propensity Score Matching (PSM) | Demographics (gender, age), Charlson comorbidity index, prior comorbidities (e.g., Hypertension, Heart failure, Renal diseases, Stroke/TIA, IHD, AF), non-SGLT2I/DPP4I medications (e.g., Beta blockers, Anticoagulants, Lipid-lowering drugs), baseline fasting glucose, HbA1c, NLR, CBCs, and liver/renal biochemical tests (e.g., Creatinine, Urea, Albumin) |
| Hong et al. (2024) | Propensity Score Matching (PSM) | Age, sex, income level, calendar year, proxies for diabetes severity (other antidiabetic drugs, number of types, treatment level), severity of mental disorders (type of disorder, psychiatric prescriptions/types, hospitalizations for mental disorders), comorbidities (e.g., diabetic complications, ischemic heart disease, hypertension, heart failure, atrial fibrillation, CKD, cancer), concomitant drugs (e.g., beta-blockers, ACEi/ARB, CCBs, diuretics, lipid lowering drugs, antiplatelets, NSAIDs), Charlson comorbidity score (CCI), and healthcare utilization |
| Zhuo et al. (2025) | Inverse Probability of Treatment Weighting (IPTW) | Demographic characteristics (age, sex, education level, married status, insurance type); BMI; duration of antidiabetic treatment; comorbidities; co-use of prescription drugs (other antidiabetic drugs, cardiovascular medications, lipid modifying agents, PPI); and healthcare utilization (visits, admissions, test frequency of HbA1c, lipid, creatinine, uric acid); Year of index date |
| Chen et al. (2024) | Propensity Score Matching (PSM) followed by Multivariable Cox Regression (for subgroup analysis) | Age, sex, underlying diseases (hypertension, hyperlipidaemia, hyperuricemia, chronic kidney disease, chronic liver disease, chronic obstructive pulmonary disease, thyroid disease, peripheral vascular disease, sleep apnoea, rheumatic heart disease), AF ablation status, and medications (anti-arrhythmic drugs, warfarin, NOACs, anti-platelets, insulin, DPP4i, ACEI, ARBs, MRAs, CCBs, beta-blockers, and statins) |
| Pai et al. (2024) | Propensity Score Matching (PSM) | Age, sex, race, ethnicity, smoking, alcohol consumption, obesity, hypertension, hyperlipidaemia, hypoglycaemia, ischaemic heart disease, atrial fibrillation, cerebrovascular disease, transient ischaemic attack, chronic liver disease, and chronic kidney disease |
| Abdullah et al. (2025) | Propensity Score Fine Stratification Weighting | Comorbidities, medication use, smoking and alcohol use, diabetes duration, HbA1c, BMI, number of physician visits, and covariates associated with frailty (falls, housebound, tremor) |
| Liu et al. (2025) | Propensity Score Matching (PSM) | Age at index, race, gender, HbA1c levels, concurrent medications, and comorbid conditions (including mood/anxiety disorders, T2DM complications, hypertension, dyslipidemia, CKD, IHD, overweight/obesity) |
| Shin et al. (2024) | Propensity Score Matching (PSM) | Over 110 baseline covariates, including: personal/sociodemographic factors, complications from diabetes, classes and number of antiglycaemic drugs, risk factors for dementia (cardiometabolic risk factors, hearing loss, head trauma, fracture history, mood/mental disorders, anticholinergic drugs), other comorbidities, Charlson-Deyo CCI, and healthcare service use patterns |

**Supplementary Table 4:** Baseline Prevalence of Key Dementia Risk Factors

| **Study (First Author, Year, Location)** | **Adjustment Method (N pairs)** | **Hypertension** | **CVD: Myocardial Infarction/IHD** | **CVD: Heart Failure (HF)** | **CVD: Atrial Fibrillation (AF)** | **Cerebrovascular Disease/Stroke** | **Chronic Kidney Disease (CKD)** | **Other GLDs: Insulin Use** | **Other GLDs: Metformin Use** |
| --- | --- | --- | --- | --- | --- | --- | --- | --- | --- |
| Pai et al. (2024) (Global, ≥50 yrs) | PSM (193,948 pairs) | SGLT2i: 81.4% Comp: 80.82% | SGLT2i: 27.63% (IHD) Comp: 27.09% (IHD) | NA (Adjusted for) | NA (Adjusted for) | NA (Adjusted for Cerebrovascular/TIA) | SGLT2i: 16.27% Comp: 15.96% | NA (Adjusted for) | Metformin used in both baseline cohorts |
| Chen et al. (2024) (Taiwan, AF patients) | PSM (810 vs. 1,620) | NA (Adjusted for) | NA (Adjusted for) | NA (Adjusted for) | SGLT2i: 100% Comp: 100% (All patients had AF) | NA (Adjusted for stroke) | SGLT2i: 1.36% Comp: 4.20% | SGLT2i: 21.6% Comp: 17.4% | NA (Adjusted for) |
| Hong et al. (2024) (South Korea, Mental disorders) | PS Fine Stratification Weighting (42,873 vs. 384,757) | SGLT2i: 69.2% Comp: 69.3% | SGLT2i: 2.8% (MI) Comp: 2.8% (MI) | SGLT2i: 6.6% Comp: 6.6% | SGLT2i: 3.4% Comp: 3.4% | SGLT2i: 9.0% (Ischemic) Comp: 9.0% (Ischemic) | SGLT2i: 1.9% Comp: 1.9% | NA (Adjusted for Insulin use level) | NA (Adjusted for) |
| Liu et al. (2025) (Global, ≥65 yrs) | PSM (80,376 pairs) | SGLT2i: 65.8% Comp: 66.0% | SGLT2i: 3.1% (Acute MI) Comp: 3.0% (Acute MI) | SGLT2i: 12.2% Comp: 11.8% | SGLT2i: 10.5% Comp: 10.3% | SGLT2i: 8.0% Comp: 7.7% | SGLT2i: 17.0% Comp: 16.6% | NA (Adjusted for concurrent medications) | NA (Adjusted for concurrent medications) |
| Zhuo et al. (2025) (China, ≥50 yrs) | IPTW (27,590 vs. 19,527) | NA (Adjusted for) | NA (Adjusted for) | NA (Adjusted for) | NA (Adjusted for) | NA (Adjusted for) | NA (Adjusted for) | NA (Adjusted for) | NA (Adjusted for) |
| Shin et al. (2024) (South Korea, 40-69 yrs) | PSM (110,885 pairs) | SGLT2i: 66.6% Comp: 66.5% | SGLT2i: 3.0% (MI) Comp: 3.1% (MI) | SGLT2i: 7.7% Comp: 7.8% | SGLT2i: 2.9% Comp: 2.9% | SGLT2i: 6.4% (Stroke) Comp: 6.3% (Stroke) | SGLT2i: 5.7% Comp: 5.8% | SGLT2i: 7.4% Comp: 7.5% | SGLT2i: 76.5% (Biguanide) Comp: 77.4% (Biguanide) |
| Wu et al. (2023) (Canada, ≥66 yrs) | IPTW (36,513 vs. 36,545) | SGLT2i: 76.5% Comp: 75.9% | NA (Adjusted for Percutaneous Coronary Intervention) | SGLT2i: 13.8% (Congestive HF) Comp: 13.8% (Congestive HF) | SGLT2i: 6.0% Comp: 5.9% | SGLT2i: 2.5% Comp: 2.5% | SGLT2i: 10.6% Comp: 10.6% | SGLT2i: 22.7% Comp: 22.7% | SGLT2i: 68.6% Comp: 68.1% |
| Abdullah et al. (2025) (UK, ≥40 yrs) | PS Fine Stratification Weighting (34,797 vs. 82,939) | NA (Adjusted for) | NA (Adjusted for) | NA (Adjusted for) | NA (Adjusted for) | NA (Adjusted for) | NA (Adjusted for Chronic Renal Insufficiency) | NA (Adjusted for) | NA (Adjusted for) |
| Mui et al. (2021) (Hong Kong, median 66.3 yrs) | PSM (13,283 vs. 26,545) | SGLT2i: 22.85% Comp: 18.39% | SGLT2i: 13.45% (IHD) Comp: 8.81% (IHD) | SGLT2i: 1.53% Comp: 0.95% | SGLT2i: 2.80% Comp: 4.98% | SGLT2i: 2.89% (Stroke/TIA) Comp: 2.32% (Stroke/TIA) | SGLT2i: 1.34% (Renal diseases) Comp: 2.68% (Renal diseases) | NA (Included in Other AD drugs) | NA (Included in Other AD drugs) |
| **Abbreviations:** NA = Data not explicitly provided or quantified; TIA = Transient Ischemic Attack; IHD = Ischemic Heart Disease; GLD = Glucose-Lowering Drug. | | | | | | | | | |

**Supplementary Figure 1.** *S*ensitivity analysis for the association between SGLT2 inhibitor use and risk of Alzheimer’s disease after exclusion of Liu et al.


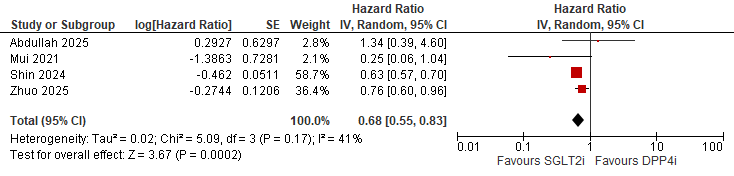

Supplement: Supplementary file 1 — Table S1: Detailed search strategy used in each database. Table S2: Quality assessment of included cohort studies using the Newcastle‐Ottawa Scale (NOS). Table S3: Key adjustment factors and covariates. Table S4: Baseline prevalence of key dementia risk factors. Figure S1: Sensitivity analysis for the association between SGLT2 inhibitor use and risk of Alzheimer's disease after exclusion of Liu et al. [file EDM2-9-e70174-s001.docx]
